# The Social and Emotional World of Twentieth-Century Anglo-American Surgery: The James IV Association of Surgeons

**DOI:** 10.1353/bhm.2022.0002

**Published:** 2022

**Authors:** Agnes Arnold-Forster

**Keywords:** surgery, twentieth century, sociability, emotions, professional identity, Britain, United States

## Abstract

Founded in 1957 by a group of elite British and American surgeons, the James IV Association of Surgeons is an international organization that "promotes communication among surgeons across the globe." Every year since 1961, the association has funded trips for several "surgical travellers" to encourage "exchange and camaraderie between surgical communities." This article uses the association's archive to explore the social lives, professional identities, and affective experiences of the men and women who populated the "surgical world" of Britain and North America in the mid-twentieth century. Integrating the social history of medicine with emotions history, I argue that the social lives of surgeons were crucial to the development and maintenance of their professional identities and communities by assisting in the definition of what it meant to be a surgeon. This definition was structured not just by surgical skill but by the forms of sociability available to potential participants.

In 1964, Boston surgeon Bentley P. Colcock wrote up his report detailing his recent trip to the United Kingdom in his capacity as one of the James IV Association of Surgeons' "surgical travellers." He wrote, "I have always felt that one of the most interesting aspects of a surgeon's life is the[Other P-71] unusual men (and women) he meets in his 'surgical world.'"^1^ Founded in 1957 by a group of British and North American surgeons, the James IV Association of Surgeons was, and continues to be, an international organization that "promotes communication among surgeons across the globe."^2^ Since 1961, the association has funded trips for several "surgical travellers" each year to encourage "exchange and camaraderie between surgical communities."^3^ These "surgical travellers" were drawn from the upper echelons of the profession and tended to be particularly ambitious practitioners who used the association to advance their careers. The trips offered surgeons a unique opportunity to travel and study, and many of the members ended up occupying influential positions. This article uses the association's archive to explore the social lives, professional identities, and affective experiences of these "unusual men (and women)" who populated the "surgical world" of Britain and North America in the middle of the twentieth century.

Integrating the social history of medicine with emotions history, I argue that the social lives and workplace cultures of surgeons were crucial to the development and maintenance of their professional identities and helped to define what it meant to be a surgeon after the Second World War. As the association's archives make clear, this definition was structured not just by surgical skill but by the forms of sociability available to potential participants and by the "character" of the men ("and women") involved. These archives provide evidence for a surgical culture and community that was actively made and maintained; and one that was primarily open to white, English-speaking men from affluent social backgrounds.

In what follows, I interrogate the ways in which surgeons' social lives—both on and off the wards—were used to demarcate professional difference.^4^ As an example of this process, this account of prominent surgeon Sir John Bruce's character attests to the importance of personality and sociability in the making of the mid-twentieth-century surgeon:

His multitudinous friends and colleagues over the globe testify to the bonds he was able to create and countless young men of all nationalities owe much to him for furthering their careers. Indeed, this was one of his greatest qualities—a sympathy for and a receptive ear to the young of all ages; though he[Other P-72] walked with the kings of his profession, and was one himself, he was the ready counsellor of successive generations of aspiring surgeons. His gregariousness and readiness of wit overshadowed to some extent his solid achievements in clinical surgery.^5^

Bruce was a social and professional engineer—lubricating the careers of his junior colleagues. His "greatest quality" was his "receptive ear"—a "king" of the profession not because of his surgical skill (although no doubt he possessed some), but because of his ability as a "ready counsellor." Indeed, the obituary made this distinction explicit. His "gregariousness" and good humor "overshadowed" his "solid achievements in clinical surgery." Bruce embodied the ideal surgeon. He possessed attributes that made him likeable, relatable, and supportive and gained him entry to the surgical community. However, his behavior and attributes also cultivated a surgical culture, one that was coded white, male, and socially elite. In this way, I argue, the praise given to him by his association colleagues, made a general statement about boundaries and the surgical profession, rather than just a specific statement about him as a man.

Obituaries like this one, as well as the travelers' reports, also allow us to better understand the emotional tenor of professional sociability and the "emotional regimes" under which surgeons worked.^6^ The James IV Association of Surgeons did not ask its members to report on what they felt while at work or abroad, but nonetheless the archive is replete with affective language. A consideration of surgeons' emotional lives is a relatively recent scholarly endeavor and has been conducted by researchers from a range of disciplinary backgrounds. Michael Brown's examination of early nineteenth-century surgery shows how the adoption of certain emotional styles was crucial for self-promotion and the delineation of the surgeon's identity and social utility.^7^ Similarly, Rachel Prentice's ethnography of anatomical dissection, surgical education, and training attests to the close relationship between emotions and professional identity formation in contemporary America.^8^ The association's archive demonstrates the[Other P-73] importance of affability and gregariousness to the mid-twentieth-century surgeon and his inclusion within this elite social and professional circle.

But emotions were also crucial to other aspects of the association's activities. In this article, I argue that members policed the boundaries of their profession and did so using emotional strategies and rhetoric. In addition, I also suggest that the surgical travelers' reports can be used to gain insight into surgeons' ideas about, and experiences of, "well-being"—however oblique. They contain debates about working conditions and overwork, feelings of calm and command, and discussions about whether their workplaces cultivated a sense of belonging and community.^9^ Thus, while the social lives and workplace cultures of midcentury surgeons were, in many ways, problematic, I argue that aspects also ameliorated the working conditions of professionals from across the British Isles and North America and protected their emotional health.

Considering its central place in the history of medicine, and the regularity with which people have operations today, surgery has not yet received the attention it deserves from professional historians, or even professional historians of health care.^10^ As a result, while the professionalization of surgery has attracted some attention from historians of the nineteenth century,^11^ we know much less about how the surgical identity was later made and maintained. Exceptions include Delia Gavrus's work on neurosurgeons, Thomas Schlich's research into fracture care, and Peter Kernahan's chapter on professional boundaries in the early twentieth century, which explores how surgery, once it was a part of the medical profession, went on to become a specialty.^12^

Kernahan delineates how surgical associations and other organizations claimed authority over the regulation of surgical practice and suggests[Other P-74] that the development of surgery as a specialty was a process of continuous definition and redefinition of professional boundaries. He outlines how the borders between different groups performing similar work were established and contested and, as Schlich puts it, argues that "the identity of surgery was not determined by the nature of things; it was an object of negotiation."^13^ This article follows Kernahan's trajectory, but veers off in a slightly different direction and attends to later in the twentieth century. Rather than exploring how a surgical association claimed authority over surgical practice, I examine the "cultural history" of professionalization and analyze a surgical society's attempts to create social connections, build international networks, and implicitly restrict the conditions of entry to the surgical community.

This article begins by situating the association in its historical context and examines the various ways the organization's participants used "the past" in their professional self-fashioning. Then, it explores the social lives and pursuits of association members before examining the relationship between surgeons' sociability and language and ethnicity, gender, and social class and salaries. This is a comparative, transnational study that looks at practitioners from Britain, Canada, and the United States and their social and professional encounters with colleagues from the other side of the Atlantic. While the health care delivery systems in each country were distinct, the association's success demonstrates that there were shared cultural values, norms, and practices that underpinned the sociability of elite midcentury surgeons.^14^ This article then turns to the emotional landscapes of British and North American hospitals, suggesting that this sociability protected the "well-being" of surgeons in the middle decades of the twentieth century. Finally, the conclusion explores the lasting impacts of these social lives and workplace cultures on the diversity of the surgical profession and on the emotional health of twenty-firstcentury practitioners.[Other P-75]

## History and the James IV Association of Surgery

Sir John Bruce, along with Ian Aird from Edinburgh and J. William Hinton from New York, were responsible for the foundation of the James IV Association of Surgery in 1957. Bruce and Aird were in Atlantic City, New Jersey, to be made honorary fellows of the American College of Surgeons and met with Hinton to discuss their plans for a new professional organization.^15^ The association's archive is held at the New York Academy of Medicine and includes minutes from meetings in 1964, 1971, 1975–84, and 1988, surgical traveler reports, and association volumes and pamphlets. In their reports, the surgical travelers recounted their trips in detail and offered observations and reactions to medical practices, facilities, and education in the countries they visited. The printed volumes include a short history of the association, bylaws, text of the certificate of incorporation, and lists of members, many of which are supplemented with biographical information. Pamphlets contain brief reports on the state and mission of the association and lists of officers, members, and travelers.

Bruce and Aird named the society the James IV Association because of the supposedly unbroken line of descent from the Incorporation of Barber Surgeons of Edinburgh, whose first charter of 1505 was ratified by King James IV of Scotland (1473–1513), to the Royal College of Surgeons of Edinburgh.^16^ Surgeons tend to venerate their own history, and association members saw their achievements as part of a long lineage of past successes and heroic figures. Surgical travelers from North America invariably devoted some of their report to drawing connections between their colleagues in Britain, the places in which they worked, and their illustrious surgical antecedents. Bentley P. Colcock was thrilled to discover, "My lecture to the students was given in the amphitheatre in which Lister had operated."^17^ Lister is a recurrent feature of surgical traveler reports, and he repeatedly appears as an impressive member of British surgeons' professional "family tree." Hugh E. Stephenson wrote,

When one realizes that Professor Sir John Bruce, as Regius Professor of Surgery at the University of Edinburgh (and surgeon to Her Majesty the Queen in Scotland), is a direct administrative descendant of Professors Syme and Lister, it is easy to understand the influence which some of the great professors of surgery have exerted in their departments (there have been 8 professors[Other P-76] of clinical surgery at Edinburgh since 1802); great continuity of purpose is allowed by such a tenure.^18^

Similarly, "Professor Sir Charles Illingworth of Glasgow is 4 generations removed from his predecessor, Lord Lister." Lister is perhaps the most famous man in the history of British medicine. Born in April 1827, he was a surgeon and pioneer of antiseptic operative practice. President of the Royal Society between 1895 and 1900, he was raised to the peerage in 1897. He is one of only two surgeons in the United Kingdom with a dedicated public monument (the other is John Hunter), and after his death in 1912, his funeral service was held at Westminster Abbey. Lister is also the protagonist in a range of popular and sycophantic biographies (many written by surgeons).^19^

In another tribute to Sir John Bruce (1905–75), Adam N. Smith alluded to his historical lineage, crucial to the individual and collective identity of the twentieth-century surgeon: "Sir John was Edinburgh's great ambassador to the world of surgery. The rich heritage of the Regius Chair of Clinical Surgery—he was in the line of Syme and Liston, Annandale, Stiles and Fraser—brought invitations to many centres where he imparted a zestful Scottish presence to many a surgical meeting."^20^ Like Lister, Robert Liston holds a prominent, if more ambivalent, place in the history and historiography of surgery. As Michael Brown has argued, his twentiethcentury reputation as a speed-obsessed showman is largely the product of a mythology whose origins can be traced back to the early twentieth century.^21^ History and heritage thus loomed large in the experience of surgical travelers to the United Kingdom. Hugh Dudley wrote that John Bruce was a "personality of some grandeur, perhaps in some ways more suited to a larger age than ours."^22^

Heritage was also present in the built environment of mid-twentiethcentury surgery. Stephenson described the presence of the past in the[Other P-77] architecture of British medicine: "While little hospital or medical school construction has taken place since before World War II and facilities are old in some instances, many are architecturally beautiful and, indeed, rich with reminders of physicians who have worked and contributed so significantly in the past."^23^ Making similar comments on a specific hospital, Eric W. Fonkalsrud wrote, "There is a marvellous charm in such hospitals as St Bartholomew's which was constructed in the twelfth century and which has beautiful Hogarth murals on the wall of one hall and beautiful brasses in the chapel, including that of William Harvey overlying his tomb."^24^ Much like many of the association's members, Fonkalsrud was an ambitious surgeon who went on to become a leading pediatric surgeon at UCLA medical center. On Colcock's first night in London, he attended a dinner at the Royal College of Surgeons as guests of Sir Arthur and Lady Porritt, "The beautiful panelling of the dining room and council chamber, the portraits of famous surgeons, and the restored Hunterian Museum and Nuffield College made a perfect setting for a surgeon's introduction to the United Kingdom."^25^ This conceptualization of British heritage—its illustrious past embodied in its architecture—should come as no surprise to those interested and invested in British national identity, its self-image, and its class politics.^26^ In elaborating on the heritage and history of the profession, association members were making and maintaining a kind of surgical aristocracy. Surgery was (and continues to be) a profoundly hierarchical community, one where seniority is respected, where individuals and interventions carry the weight of their ancestors, and where professional positions were as much inherited or bestowed as they were earned. This was particularly true in the United Kingdom, and visitors from North America took the opportunity to revel in, rather than rebuff, this British ideal.

In postwar Britain, surgeons trained as part of a "firm"—a hierarchical structure of senior and less-senior practitioners. The "firm" orbited around both the hospital ward and the hospital bar. In a 1969 survey of medical staff at Northampton General Hospital, the junior doctors blamed[Other P-78] "the firm" for several of their complaints. For example, they lamented the nepotistic nature of promotion decisions and advocated for a new career structure that "might allow an optional system of staff reporting as used in the Civil Service" because it would "place more emphasis on the overall record of the doctor when an appointment is being considered and would reduce the influence of a single consultant which is so strong under the present system."^27^ The ideals and practices of patronage and lineage were strong in "the firm" system. In British surgeon's T. C. Graves's obituary, published in 1964, the first few sentences identify him as "the grandson of the founder of the firm of Chivers."^28^ For some, "the firm" connoted prestige and a kind of surgical "family"—complete with ancestors and descendants—for others, it made career progression and opportunities for promotion dependent on the personality of sometimes capricious individuals.

## Sociability

The firm was, therefore, as much a social organization as a unit of work. In similar ways, the James IV Association blurred the boundaries between the personal and the professional. As Peter Kernahan argues, surgical associations and societies claimed increasing authority over the regulation of surgical practice and defined intellectual boundaries both between surgery and other clinical fields, and between distinct surgical subspecialties.^29^ The James IV Association served a slightly different purpose. Rather than a gathering of minds to explore the latest surgical problems or present the latest surgical innovations, and much like earlier intellectual societies, the association offered a gathering of personalities designed to ameliorate the social lives and lubricate the social exchange of mid-twentieth-century surgeons.^30^ Colcock, writing in 1964, made this distinction clear:

If I have seemed to emphasize men, the surgeons themselves, over surgical principles and surgical technique, it has been done deliberately. One does not need to cross the Atlantic to learn the English, Scottish or Irish approach to various surgical problems, but it is only by personal contact that we can fully[Other P-79] appreciate the men who have written these articles—their experiences, their vision, and their intellectual honesty.^31^

Indeed, the suggestion that the annual meeting in 1977 (held in Dublin) should be an all-day meeting, with the usual lunch but on this occasion preceded by a presentation of scientific papers in the morning and followed by a research outing in the afternoon, was met with concern. Dr. Loyal Davis stated that while he did not want to influence the members, he would vote against the scientific papers being presented as it was "his understanding that the the [*sic*] Association would, at no time, have a presentation of papers, as the Founders and Members wanted the Association to be unique and not follow the custom of other surgical associations in this matter."^32^ Davis's objections were sustained, and no scientific papers were presented—establishing the association's identity as a place of relaxed sociability rather than formal intellectual exchange.

This connection with relaxed sociability was repeatedly confirmed by the travelers' reports. An emphasis on eating and drinking permeates the association's archive. Meals were key to the construction of socialsurgical relationships and the primary purpose of association-sponsored travels abroad. In his undated report on his trip to the United Kingdom, Ireland, Denmark, and Sweden, R. A. Macbeth, from Alberta, Canada, wrote, "Not once did I have lunch alone, nor did Monique [his wife] and I have dinner alone while in Glasgow—and this pace kept up for virtually the entire trip."^33^ Colcock devoted much of his report to a description of the food and alcoholic beverages he consumed while traveling around Britain: "After stopping at the [Andrew] Kay home to see the children and collect Mrs Kay, we had dinner at a delightful village inn outside the city. The excellent food, fine wine, and much good talk with two wonderful people made it a perfect evening."^34^ Sometimes these descriptions were little more than lists of different drinks: "The martinis, the burgundy, the scotch, and Drambuie, interspersed with excellent food and the sparkling personality of John Bruce, made it a grand evening."^35^

The traveler report was an informal genre of writing, and surgeons evidently felt comfortable focusing on their social and culinary encounters.[Other P-80] Some even described less salubrious experiences: "I apologize that my account will not be as eloquent as many of those I read. However, I wrote my report each evening during my trip unless the evening festivities resulted in a mild, to occasionally middling, alcoholic haze!"^36^ Perhaps unsurprisingly for anyone who has attended professional events in both places, descriptions of alcohol consumption were more frequent in the reports by travelers who visited the United Kingdom than in those who journeyed to North America. Folkert O. Belzer from Wisconsin traveled to England in 1977. He wrote, "At the hospital, we first had lunch with the entire staff. … We had a delightful lunch including a glass of sherry and a good Bordeaux wine with the meal, a habit we should introduce in the States!"^37^ On his later trip to Poland, Belzer continued his forensic exploration of local intoxicants: "We tasted the local Polish drink, slivovitz, which I must say has a rather potent and delayed action."^38^

The consumption of food and drink was, of course, always accompanied by conversation and social exchange. The surgical travelers reported on both fine dining and warm hospitality. Writing in 1961, E. G. Muir from London, described his trip to Canada and the United States: "My wife and I received everywhere the most charming and generous hospitality, a hospitality indeed which frequently makes the recipient feel quite unworthy of it—but determined to return for more!"^39^ In a similar vein, Edward G. Tuckwell described his 1963 travels in Canada: "From Montreal to Edmonton … were wonderfully entertained by Walter Mackenzie, Bob Macbeth and Walter Anderson and their wives."^40^ While British surgeons were more likely to lubricate the lunches and dinners of their guests, North Americans were no less welcoming: "Americans are, of course, famous for their hospitality and the surgical traveller from Ireland was liberally exposed to this delightful phenomenon. My wife and I are most grateful to all those who were so kind to us."^41^ The personalities of the various surgeons whom different travelers encountered were, therefore, key to their overall impression of their trip.[Other P-81]

Traveler reports, like this one written by Colcock, contained lengthy descriptions of the character and kindness of the surgeons they encountered:

Sir Arthur Porritt who made that evening a memorable one for my wife and me. Warm and friendly, he soon made us feel completely at ease even though we were among strangers. His alert mind, his intuitive "feel" for others, and his sincerity, add up to a personal magnetism not given to many men.^42^

This emphasis on personalities and social interactions was occasionally made explicit. Bentley P. Colcock described walking back to his hotel with his wife who "informed me that Sir Arthur [Porritt] had told her that I was 'not here to work, but just to meet people'—a delightful assignment!"^43^ There was an emotional quality to this sociability too. These surgeons derived pleasure from their work and experienced joy, warmth, and connection. This distinction between work and pleasure did not, however, usually apply to the surgical career. Much like the surgical community at large, the association defined pleasure *as* work and blurred the boundaries between personal and professional lives. British doctor Ronald Macbeth described hospital clinical work as "quite literally full-time."^44^ He advocated for the traditional practice of trainee surgeons living on the hospital site by insisting on the professional and educational value of the opportunities for informal exchange that being a hospital *resident* produced, such as eating together. He wrote to the *British Medical Journal* in 1963, "Being around in the mess for the casual discussion of cases and for consultation at resident level … these are the stuff whereof the training of a good doctor is made."^45^

When assessing surgeons for potential new membership, the association relied on social characteristics as much as on clinical aptitude or interest—whether recruits were well *liked* was crucial. If possible members had previously entertained past surgical travelers, then they were more likely to be looked upon favorably in assessments of their character. For example, "Lesley Harold Blumgart … has entertained several Surgical Travellers and is an excellent candidate for active membership" and "Geoffrey R. Giles … is a strong chap has entertained previous Travellers and would[Other P-82] make an excellent member."^46^ This description of potential members as a "strong chap" was a common feature of proposed association participants. Alan G. Johnson was also "a very strong chap."^47^ The ways in which surgeons conducted themselves in social interactions became a crucial aspect of the assessment of whether they "belonged" in the profession. To participate in this community, surgeons had to be able to cultivate a certain emotional and sociable experience for their colleagues. In this way, the association also served to articulate and draw boundaries around who constituted a surgeon and determined who was allowed entry to the social world of mid-twentieth-century surgery.

## Language and Ethnicity

This boundary making extended beyond personality and character to incorporate discussions—implicit and explicit—of race, ethnicity, and language as determining aspects of someone's suitability for inclusion in the society and as conditions of entry to the surgical profession. Colcock ruminated on the lofty ideals of surgery and considered the grand designs of the association: "Since my experiences as a surgical traveller, I am more than ever convinced that we as physicians have a unique advantage in helping to solve these problems."^48^ This "unique advantage" was their capacity—"unlike any other profession in the world"—to speak a "universal language." Like his fellow travelers, he emphasized character alongside surgical skill: "Wherever we are, whoever we are, our main interest in life is to help our fellow man. It is our privilege and our duty to serve wherever and whenever we are needed—not only with our hands and our minds—but with our hearts as well."^49^ The "universal language" he referred to was the lingua franca of scientific humanity. In contrast, the association was far more specific and restrictive in its determinations of the *actual* language its members should use to communicate and connect. There were plenty of differences between Britain and North America, but they had enough in common—linguistically and culturally—so that surgeons could connect and share similar forms of sociability. Thus, while the travelers might have advocated for a broad, international community of surgeons, in reality that community was geographically circumscribed.[Other P-83]

The minutes of a 1979 meeting of the North American members record a discussion of potential travel destinations: The secretary reported "that the question had arisen as to whether a traveler should travel to a country where … the English language is not freely spoken."^50^ This came about after one of the recent travelers had gone to China where there is not a James IV Member and interchange was carried out through an interpreter.^51^ The members concluded that this "did not comply with the original aims of the Association, which were that the Travellers would travel to member countries where the English language is freely spoken." It is unclear why "interchange carried out through an interpreter" proved so troubling to the association or why "embarrassment" might have resulted from "the Traveller who arranges his own itinerary and makes his own arrangements without consulting the Secretary."^52^ Evidently, the association was concerned by any potential loss of control over the activities of their members. Moreover, this anxiety was prompted not by the traveler's occasional extravagant socializing, but instead by a member who journeyed beyond the Anglosphere. The association agreed that in the future, travelers should "submit his proposed itinerary to the Secretary or Assistant Secretaries for their approval before any definite travel plans are effected."^53^

While most travelers visited Europe and North America, some were permitted to venture further afield. For the association's purposes, India was considered part of the Anglosphere (English was and continues to be one of the country's official languages). When reflecting on their trips to India, some travelers made Orientalist observations and reaffirmed their commitment to English as the lingua franca of surgery. British member Victor Riddell wrote in his 1963 report,

I had never been to India. It was therefore with much pleasure that I heard of my good fortune in being nominated as a James IV Surgical Traveller. It seemed desirable that I should promptly refresh and improve my knowledge of this ancient land of "dusky faces with while silken turbans wreathed" (Milton: Paradise Regained, IV, 76).^54^[Other P-84]

He went on to praise the pluck and determination of Indian doctors: "In spite of all the difficulties and obstructions there is still a touching eagerness amongst the young there to seek knowledge overseas. Their motto, very properly seems to be: 'Travel is the life blood of medicine.'" Finally, he turned his attention to the adoption of English as an official language (in 1950): "The consequence of this decision in the political field are immeasurable."^55^ He applauded the end of "the wasteful situation in which scientific books were being translated into Hindu or Urdu" and decried the slow, painstaking, and expensive process that rendered the outputs useless and out of date.^56^

Riddell's praise of Indian doctors' travel, determination, and use of the English language was not taking place in a vacuum. Indian doctors were emigrating to Britain in increasing numbers and playing a crucial role in the new National Health Service (NHS).^57^ Despite their services to the welfare state, these doctors were subject to sometimes vitriolic attacks about their perceived ability to speak the English language and held to a higher standard of conduct and performance than their white colleagues. In 1976, Mr. P. Harding wrote a letter to the chairman of the Royal Commission on the National Health Service to complain about the foreignness of some of his health care providers: "I am particularly concerned at the quality of the administrative, financial and personnel staff employed with the NHS." His understanding of "quality" was informed by his racism:

I have a specific question concerning the medical competence and, more significantly, ability to speak the English language of foreign-educated doctors … who escaped the recently imposed set of English tests as they were registered here in the period before the recent crackdown on low grade "doctors" from abroad.^58^

He referred to two specific doctors in his letter. Despite not being a patient of either, he had serious concerns about their ability to practice and cast aspersions on their medical credentials: "I know that on a number of occasions patients … have expressed their difficulty at being able to communicate in English with (1)." He went on, "I doubt both his English language and medical competence. Just what does one make of MBBS[Other P-85] Bihar? Is it up to English standards? In the case of (2) I would also like your view as to the standard of MB Calcutta."^59^

In his 1964 assessment of undergraduate surgical teaching in the United Kingdom and Ireland, Stephenson explained why doctors from India were a crucial component of the British health system. While approximately sixteen hundred students graduated from the twenty-six medical schools each year, "this total is considered inadequate for providing proper medical care under the National Health Service." Thus, approximately four thousand (about 50 percent) of the residency positions in the British Isles were "currently filled by graduates from foreign medical schools." To partially address this shortfall: "Two new medical schools are, I understand, in the planning stage and possibly will be located at Southampton and Nottingham."^60^

The situation in the United States was similar. Under the Hart-Celler Immigration and Nationality Act of 1965, the United States began to solicit foreign medical graduates largely from South Asian nations. These graduates were granted permanent residency or U.S. citizenship in exchange for medical service in marginalized communities. Although this arrangement was conceived as a temporary solution, in the past fifty years, and much like in Britain, it has become a "permanent fix," with foreign physicians composing a quarter of the physician labor force.^61^ As in Britain, and according to historian Eram Alam, "the care provided by foreigners was received as different, an imperfect facsimile of their US counterparts."^62^ Sasha Mullally and David Wright tell a parallel story about the diaspora of trained health personnel from South Asia to Canada in the mid-twentieth century, which formed part of a large-scale migration of doctors relocating across national boundaries over the 1960s and 1970s. These international[Other P-86] movements attracted concern, not just from members of the James IV Association. As Mullally and Wright report, Alfonso Mejia, chief medical officer of Manpower Systems for the World Health Organization, noted in 1978 how "anxiety evoked by migration was reach[ing] a peak in both major donor and recipient countries." This "brain drain" was predicated on global economic inequalities and proved profoundly profitable for countries like Britain, the United States, and Canada.^63^

In all three countries, South Asian–trained surgeons and physicians brought considerable expertise, met a crucial need, and propped up their respective health care systems. While some of the association's members acknowledged the value and contributions of these doctors, and recognized that their countries, citizens, and colleagues depended on their clinical labor, the organization's uncertainty about expanding the geographical and linguistic boundaries of their surgical world served to exclude doctors from South Asia from full participation in the professional community. While the language used by some surgical travelers was Orientalist and Othering rather than explicitly derogatory or discriminatory, South Asian doctors faced substantial abuse and aggression in Britain and North America. This, coupled with the subtler exclusionary language and boundary drawing used and conducted by their white colleagues served to demarcate ethnic lines around communities of clinical professionals and cultivated both explicitly and implicitly hostile working environments for surgeons of color.

## Gender

Race, ethnicity, and language were not the only considerations the association made when defining the mid-twentieth-century surgeon. While members never explicitly prevented women from joining either the association or the profession, gendered observations featured heavily in the organization's minutes and reports. Women were praised as wives and secretarial support. In 1964, Stephenson from Missouri wrote his report of his travels to the United Kingdom and Ireland. He said, "The truism that 'behind most great men there is a woman' applies here. The wives of the professors and surgical teachers are most charming and their interest in things surgical is apparent. Some of the most pleasant evenings I have[Other P-87] ever spent were with the surgeons and their families."^64^ Women were social supports to their surgeon husbands. While they might have an "interest in things surgical," their utility did not extend far beyond charm and the provision of "pleasant evenings." Colcock agreed with Stephenson when he described a trip to Rye in Sussex, England, with another member of the association, Andrew Monro: "The young women made charming partners, the older women an appreciative audience, and, refreshed by plenty of tea and cake, it made a very pleasant interlude."^65^

Male surgeons were praised for their well-chosen spouses: "Sir Ian [president of the Royal College of Surgeons of Ireland] has another tremendous asset in Lady Fraser, a very attractive and intelligent woman."^66^ Male surgeons also praised their own wives and expressed gratitude for their company. In his summary of his visit to England, Scotland, and Ireland in 1964, Colin C. Ferguson, wrote, "My wife accompanied me on the trip and to her I shall always be grateful for her enthusiasm and support during the entire trip and particularly during the many social gatherings which were so hospitably arranged for us at each centre."^67^ Sometimes wives offered more than just social support: "My wife accompanied me throughout my travels and I felt that this was a great advantage since I am not very good at washing my own shirts."^68^ Occasionally women's intellectual and emotional labor was paid: "Behind the more effective and efficient professors of surgery one generally finds, as one might expect, a secretary of the highest calibre. Many have devoted much of their life to being surgical secretaries; consequently their value and effectiveness is difficult to measure."^69^

There was no explicit critique of female surgeons in the archive, and the association inducted its first female surgical traveler in the late 1980s. The minutes of the 1988 meeting recorded the vital debates surrounding that milestone:

Mr John McFarland pointed out to the Membership that past surgical travellers had always been male and had received neckties to wear during their traveling[Other P-88] fellowship. What would be appropriate to present to Dr Elizabeth MacKinnon (Canada), our first female surgical travellers? It was a point well taken by the membership. After a lively discussion all agreed that a gold pin with the head of James IV was the best suggestion.^70^

Of course, women qualified and practiced as surgeons in twentiethcentury Britain and North America.^71^ After the Second World War and the foundation of the NHS in 1948, and amid wider social pressure to provide equal rights to women, female participation in the labor market expanded. The need to grow the numbers of doctors was met by an increasing number of women doctors from the 1960s onward. Yet, even in the 1970s and against the backdrop of women's liberation movements, the surgical profession remained implicitly and explicitly hostile to female practitioners on both sides of the Atlantic. Indeed, according to Hugh E. Stephenson, in some British medical schools "girls" constituted one-fourth of the class.^72^ However, as in the United States, "many women graduates never practice medicine."^73^ The association's archive offers some answers to the question of why many women left the profession shortly after completing their studies.

Mid-twentieth-century surgeons cultivated a masculine culture that coded certain behaviors as appropriate and necessary for full participation. In doing so, they implicitly excluded women from the surgical world. Stephenson described the social and professional pursuits of Professor Sir Charles Illingwoth of Glasgow, who "may be found leading a group of medical students and members of his 'firm' on hikes over the rugged west highlands of Scotland." The week before Stephenson arrived, "this group had stopped to swim in a cold mountain stream."^74^ This culture was by no means confined to the James IV Association and debates in the 1960s about whether hospitals should provide quarters for married staff members that could accommodate their spouses were marked by their consistent assumption that surgeons and physicians were male. P. A. Johnson wrote to the *British Medical Journal* in 1967, "A married man wishing to continue in the hospital service cannot apply for more than 25%[Other P-89] of the junior hospital posts advertised if he wishes to remain living with his wife. Why should a man have to choose between leaving his wife or pursuing his profession?"^75^

The attitudes of surgeons toward the women who made it into the workplace (mid-twentieth-century nurses were almost exclusively female) were similarly restrictive. Stephenson recorded the tradition of Edinburgh medical students signaling their approval of lectures with "a stomping of feet." His "first encounter with this action" took place prior to the start of a lecture, "when Sister McLeod, an attractive young nurse in charge of Professor Sir John Bruce's ward, suddenly walked into the classroom accompanied by thunderous stomping of feet."^76^ It is not clear whether the students were applauding Sister McLeod's skill and technical abilities, or whether they were showing their appreciation for her "attractive" appearance. On his visit to England, Scotland, and Ireland in 1964, Colin C. Ferguson praised the proficiency and professionalism of British and Irish nurses: "Nursing Sisters in charge of surgical wards occupy a much more responsible position than do their colleagues on this side of the Atlantic."^77^ His praise contained, however, a comment on their looks:

All of them were extremely competent, pretty, young ladies with a sense of humour which certainly helped them to cope with their numerous clinical problems. They worked long hours, arranged for the admissions and discharges of patients, changed dressings, removed sutures, reassured anxious parents and gave instructions for postoperative home care.^78^

Nurses were often objects of desire in the mid-twentieth-century hospital, and they frequently married the surgeons and physicians they worked with. The 1960s witnessed the emergence of an increasingly sexualized stereotype of nurses, partly a product of popular culture like the television series and film *Doctor in the House* and the *Carry On* films. Romance novels also increasingly featured doctors and nurses as the heroes and heroines in their narratives of heterosexual love. In the 1962 novel *Staff Nurses in Love* (written under the pseudonym Hilda Pressley and published by Mills & Boon), the heroine's best friend Brenda says, "For every one Florence Nightingale in nursing … there are dozens more like me who[Other P-90] take up nursing because they think they might be able to hook a famous doctor or surgeon."^79^

This strategy was often successful—and not just in the fictional world of medical romance. Health care professionals still tend to marry one another. Forty percent of doctors today are currently married to other doctors or health care professionals, and back when female surgeons and physicians were less common, medical men married their nurses.^80^ For many reasons, therefore, nursing was an attractive career for workingand middle-class women seeking work, love, and upward social mobility. However, together, these attitudes and cultural representations served to define surgery as a male pursuit and identify women in clinical settings as either useful helpmeets or sexualized objects of desire rather than respected coprofessionals.

## Social Class and Salaries

Unlike nursing, however, surgery did not offer the same social mobility to its participants. This was mainly because those who joined the profession were usually already at the top of society's economic hierarchy. The assumptions that association members were drawn from the upper echelons of British and North American society and that their social lives would reflect that shared background pervaded the organization's archive. References to social class and economic status were both explicit and implicit. In his undated report on his visit to the United States as a James IV Surgical Traveller from Ireland, Terence Kennedy wrote, "Most British surgeons in their forties have too many heavy financial responsibilities, notably the education of their children, to be able to undertake a visit to America without considerable help."^81^ Between 1963 and 1978, the proportion of pupils at independent [private] schools in the United Kingdom fell from 8.1 percent to 5.7 percent.^82^ Thus, the "heavy financial responsibilities" shouldered by British surgeons was not shared by most of the population, suggesting that surgeons occupied a narrow, wellpaid stratum of society. In 1975, John Peter Minton traveled to England,[Other P-91] Scotland, and Ireland from Ohio. He, too, commented obliquely on the substantial salaries of British surgeon, "I admire the dedication and selfsacrifice of the surgeons I met who dutifully accept the rigors of their profession, the economic restrictions of the National Health Service and the national income tax rate."^83^

In 1975, the full-time salary of a consultant (attending) averaged between £7,536 and £10,689 (£57,506.46 to £81,566.69 today).^84^ In 1974, the top rate on earned income was raised to 83 percent (the highest permanent rate since the war); however, this applied only to incomes over £20,000 (£204,729 as of 2018).^85^ In contrast, the average gross weekly earnings of full-time manual workers in the United Kingdom was £48.63 (an annual salary of approximately £2,500).^86^ Minton went on to applaud the British surgeon for continuing to work under such trying circumstances: "The bright hope for the future is, of course, the oncoming physician who … has a major sense of responsibility to the National Health Service even when the practical realities of limited funds to support the growing demands of their family becomes apparent."^87^ Surgeons' "limited funds" were vast in comparison to other British workers, and Minton's observations suggest that his North American colleagues had even higher salaries. Indeed, as Eric W. Fonkalsrud, who visited Britain from California in 1971, wrote, "Although the average salary earned by a Consultant in Britain is considerably less than that earned by a comparable specialist in the US, of great significance is the fact that Consultants still rank in the top 5 to 9 percent of income earners."^88^

However, class did not just appear in discussions of salaries and taxation. Instead, background, culture, and the influence of upbringing on the social lives of surgeons were threaded throughout the fabric of the surgical travelers' reports. Surgeons they met on their trips were identified as much by their hobbies as by their specialties. These hobbies and leisure activities were, almost invariably, bourgeois exploits and reflected the ethnicity, wealth, and social class of mid-twentieth-century medical professionals. Stephenson visited the United Kingdom and Ireland from[Other P-92] his home in Missouri. He described the habits of the surgeons he met: "Few get involved in community civic work, but many enjoy and pursue their hobbies with great vigour. Professor Charles Wells (Liverpool) and I spent one Saturday afternoon looking over about 1500 of his young pheasants, which are being readied for the fall shooting."^89^ In Britain, pheasant shooting is a hallmark of upper-class society.^90^

Others, like the thoracic and cardiac surgeon Mr. Andrew Logan, were "representative of a sizable number who spend spare time in the serenity and beauty of a garden."^91^ Mr. Thomas Wilson, an ophthalmologist from Dublin, was an "accomplished landscape and portrait artist."^92^ The new president of the Royal College of Surgeons of Edinburgh, Mr. J. J. Mason Brown, "gave … [Stephenson] a lesson in golf (and a sound trouncing) at St Andrews Old Course."^93^ These hobbies were either expensive and therefore exclusive or a part of a culture predicated on the sociability of a certain social class. Participation in these leisure activities was a marker of inclusion into the surgical community and acted as a boundary line between surgeon and nonsurgeon. Moreover, many of these activities (shooting and golf, for instance) were coded male and therefore doubly exclusionary. In his prescriptive volume, *The Surgeon's Craft*, published in 1965, Hedley Atkins described the "typical surgeon": "He is often good at or fond of games and sports. Amongst my surgical friends, I can call to mind quite readily an Olympic medallist and three international Rugby players."^94^

The forms of sociability surgeons were expected to participate in—shooting, fishing, golf—also help explain their gatekeeping around language and ethnicity. The hobbies that they enjoyed and connected with their colleagues over were part of Anglo-American culture and so served to exclude practitioners from different social or ethnic backgrounds. The power and salience of this kind of sociability are further emphasized by interviews with surgeons of color and female surgeons (and, of course, female surgeons of color) who refer, almost without exception, to the continuing influence of surgery's "old boys' club." By this they mean the[Other P-93] community of senior, white, male practitioners who often attended the same medical schools or trained at the same hospitals and who have close personal and professional bonds with one another. Surgeons who come from outside this "old boys' club" refer to its exclusive nature and the negative impact it had on their career progression. The James IV Association was the tangible embodiment of this "club."

## Emotional Support

The workplace cultures these surgeons created and maintained excluded women, people of color, and men and women from less affluent backgrounds. However, they also shaped the professional experience for those who *were* included. These cultures and attitudes normalized and mandated a sense of duty, an uneven "work-life balance," and excessive temporal commitment. As I have suggested, surgeons considered their profession to be a literal "full-time" job. However, the "full-time" nature of the job varied according to geographical location. Surgical travelers journeying across the Atlantic in both directions commented on the different surgical styles, social lives, and workplace cultures in their respective destinations. In general, North American surgeons seemed to work longer hours than their British counterparts. As Stephenson observed, "The fact that American hospital surgical floors are buzzing with students and residents at 6:30 or 7:00 o'clock in the morning is difficult for our British colleagues to visualize."^95^ He concluded that "they [medical students] probably work less hard" in the United Kingdom than in the United States.^96^ This comparison was echoed by Alan M. Clarke, who traveled from New Zealand to North America in 1970: "Like all visitors to the United States and Canada, I was greatly impressed by how hard everybody works."^97^

The tendency of North American surgeons and their students to work harder than their British and Australasian counterparts was not, however, necessarily praised by professionals on either side of the Atlantic. Stephenson commented, "It appeared to me that the British have a knack of utilizing their time in a more efficient manner than is commonly true here, partly as a result of the elimination of much detail work."^98^ Surgeons[Other P-94] in Britain were, according to Fonkalsrud, more resourceful and strategic: "Consultant surgeons do not appear to be as burdened with the exhaustive paperwork and hospital administrative committees which are so commonplace in the US."^99^ Much of the labor that surgeons in the United States had to do—the "exhaustive paperwork" and "detail work"—was, in Britain, performed by nurses. As Adam N. Smith observed, "The [American] residents and interns do many procedures which are entrusted to the nursing staff in British hospitals; they work very long hours, and there is a competitive attitude in all phases of their training."^100^ In a similar vein to Stephenson, Smith critiqued the efficacy and efficiency of his North American colleagues: "I felt, however, that the long hours worked were in some part the result of failing to do as much per hour as is done in our own hospitals, and the result of sharing, or at least overseeing duties which at home are performed by the nursing staff."^101^

In addition, the fact that surgeons in Britain did not work such long hours as North American practitioners did not appear to affect their ability or surgical success. As Stephenson suggested, "Fortunately for the patient, and regardless of the system of medicine, most surgical procedures continue to be performed by able, sincere, entirely ethical and compassionate physicians."^102^ Of course, that they were writing about fellow association members made it unlikely that they would be critical of their colleagues' capabilities or skills. Fonkalsrud, who visited Britain from California in 1971, wrote, "Relatively few patients in Britain appear to believe that they receive inadequate health care, nor do physicians indicate that any segment of the population is receiving poor health care."^103^ Moreover, there were distinct positives to be found in the different work ethic of British surgeons in the eyes of their American and Canadian colleagues. Fonkalsrud reported, "The physicians in general are less intense than in the US and usually lunch and frequently have tea or sherry together."^104^ As Stephenson observed when he visited from the United States, this sociability and informality also permeated the British operating theater, "There is almost always a friendly and courteous atmosphere in the operating room, with seldom a display of impatience and rapport between the anaesthetists[Other P-95] and the surgeon is enviable."^105^ Community cut across lines of seniority: "Students are … often invited to have dinner with the professor and his wife at some time during their membership in 'the firm.'"^106^

The most positive effect of British working culture was on the experiences, and even emotional health, of mid-twentieth-century surgeons. Stephenson described his British counterparts: "These men appear to live well-organized lives. They are less frantic, more relaxed, and seem more self-assured than their surgical colleagues on this side of the ocean."^107^ This idea that the culture and community of British hospitals improved the working conditions of British surgeons was widespread and commented on by both British and American travelers. In 1963, Edward G. Tuckwell visited the United States from his home in London, England. In his comparisons of the two health care systems and differing medical practices, he wrote, "Our 'firm' system does give the students a good team spirit and opens the way to instruction in all aspects of surgery by a large number of instructors."^108^ This notion that the "firm" offered a sense of belonging and community was widespread and can be found in other discussions of British surgical life.

The "firm" offered intangible and affective benefits to its members. In his 1968 speculative account of hospital life, chaplain George Day described what he would do if he wanted to find out about the health and well-being of junior doctors,

I would make a point of lunching and dining quite frequently in the resident house doctors' mess, and keeping my ears open. I would even drop in for a latenight beer or coffee—or even lose half a crown to them at their poker school. In this way, I would come to learn more about the … things that matter—that is, the personalities, the clash of personalities, and the fluctuating morale. For these young chaps are in the front line of the battle, holding the fort during the hours of darkness and at week-ends, when their chiefs are away.^109^

In this description, he alluded to the affective value of the firm and clinical community. He identified informal interactions, the intangible exchanges, the subtle texture of working life as "the things that matter." The various aspects of the surgical community this article has discussed—the cultivation of certain cultures and the drawing of professional boundaries along[Other P-96] gender, class, and racial lines—served to bolster the "things that matter" and produce a cultural homogeneity that helped participants feel like they belonged. This sense of belonging also served to protect surgeons from the stresses and strains of surgical life and the excessive temporal commitments their jobs demanded. As Day suggested,

Many housemen [trainee surgeons and physicians] go through a phase of deep despair; despair that they will ever get on top of their job; that they will ever give satisfaction to their sometimes thoughtless and exacting chiefs; despair that they cannot afford their patients all the unhurried attention they once hoped to be able to give. They often become exhausted in body, mind, and spirit.^110^

His solution to this mental and moral strain was friendship: "They need befriending. They need to be reassured that they are doing a fine job—as fine a job as anyone could do in the circumstances. They need their spirit renewing within them—to be made to feel worthwhile."^111^ In similar terms, a pediatric surgeon I interviewed in 2018 about his earlier experiences of surgical life attested to the supportive nature of this team approach and argued that he could cope with excessive working hours because he was maintained by a sense of comradery. This surgeon was born in 1944 and worked from the 1960s onward. He lamented the loss of this "family-like" structure, "I am quite happy that I lived through that old era." In the "old system … you felt very much like you were part of a firm. … Whatever you were doing … you were definitely working within the team and that gave a very strong feeling of belonging and commitment."^112^ He developed close relationships with his colleagues and superiors, who could rely on him: "I think if I look at all the surgical bosses I had I think I worked well with them because I was sort of around all the time and they got to trust me."^113^

There were positive and protective aspects of North American working culture too. Adam N. Smith, in his 1963 trip from Edinburgh to the United States, wrote, "I found it both impressive and exhilarating to meet the American medical teacher and his students. … They live in a much freer system and are less tradition minded than in our country."^114^ He praised the role of the medical student as part of the "team" and as a "junior colleague": "He feels he is part of a closely-knit system, and that his[Other P-97] opinion, no matter how junior, is always attentively listened to and given special consideration."^115^ This culture of respect had a beneficial impact on the experiences and practices of U.S. medical students: "Apathy in the student ranks was certainly missing in this School [Western Reserve]. The sincerity and enthusiasm of all the teaching staff met in this School was impressively high."^116^ He went on to say, approvingly, "It was always noticeable how little formality there is between higher members of staff and students."^117^ He also, however, suggested that American medical schools had to undertake a slightly different approach to cultivate the sense of community fostered by the British firm system: "In the newer medical schools an obvious attempt is made to produce a corporate spirit and enthuse people in many aspects of their life in hospital and university medical school."^118^

Unlike in Britain, where cultures, traditions, and long-standing surgical identities produced a sense of community and mutual support, hospitals in the United States had to work harder to cultivate a similar emotional landscape. Some clinical and education institutions had to make deliberate use of newspapers, journals, and published reports to foster "team spirit." Elsewhere, hospitals and universities deployed awards and public achievements to cultivate commitment. On his tour of the United States, Smith was "amused to see in one library in a very new school a plaque recording an achievement of a member of staff which ended with the words 'he surpassed the call of duty.'"^119^ The observations of surgical travelers when they crossed the Atlantic implied that British hospital cultures were the product of ephemeral, intangible, even "natural" aspects of surgical identity, training, and habits. In contrast, American hospitals and health care professionals had to take a more "artificial" approach and curate a sense of belonging and commitment on behalf of their medical students and surgeons by deliberate acts of community building. In doing so, these men suggested that hospitals' ethnically homogenous, socially elitist, and macho cultures were themselves "natural" and inevitable, rather than constructed and actively maintained by individuals and professional organizations like the James IV Association of Surgeons.[Other P-98]

## Conclusion: Beyond the Twentieth Century

This article has used the James IV Association of Surgeons' archive to explore the cultural history of surgical self-fashioning. It has identified the subtle but pervasive ways in which the surgical identity was made and maintained in the middle decades of the twentieth century. I have argued that this group of elite practitioners policed the boundaries of their community and caste through subtle and explicit sexism, xenophobia, and classism. Crucially, there was also a specific emotional content in these efforts to delineate the profession's borders. The exclusion of women and people of color from the surgical community was framed not—in this instance—in terms of skill, ability, or aptitude, but instead around what they could or could not offer to the pleasures of professional life. Success and social inclusion were much more about how people felt in each other's company, their ease of communication and collaboration, and some ineffable sense of belonging and community.

I also tentatively suggest that the social lives and workplace cultures of mid-twentieth-century surgeons have had a lasting impact on the diversity of the profession, which remains largely populated by white men from relatively wealthy social backgrounds.^120^ While much about surgery and the world it inhabits has changed, research suggests that race- and gender-based discrimination is widespread in the twenty-first-century profession.^121^ Structural barriers remain—including those that prevent parents (and mothers specifically) from career advancement—but female surgeons and surgeons of color interviewed today also attest to cultural obstacles and a "hidden curriculum" that excludes them from access to, and full participation in, the professional community.^122^ This article has demonstrated the deep roots of these obstacles.

My analysis has also historicized current and fretful conversations about the emotional health of surgeons. Anxieties about their working conditions are widespread, and their "well-being" is increasingly the target of institutional, regional, and national policies—on both sides of the[Other P-99] Atlantic.^123^ Discussions of stress, burnout, and overwork among surgeons have become ubiquitous in recent years, and the emotional cultures of hospitals are increasingly a priority for professional societies and health policy makers alike.^124^ However, as these organizations and governments (at least in the case of Britain) devote time and resources to improving the working lives of surgeons and other health care professionals, there has been little consideration of the historical context of surgical work.

Finally, this article has also shed light on the protective nature of sociability in Britain and North America, arguing that it potentially ameliorated surgeons' emotional health. This raises both challenges and possible opportunities for practitioners and policy makers eager to improve the well-being of hospital staff today. For example, if the solution to emotional distress is friendship, comradery, and a sense of belonging, then that problematizes current efforts to improve well-being through individualistic interventions such as resilience training or staff yoga classes.^125^ It also calls into question current campaigns, particularly in Britain, that attempt to improve the emotional health of hospital staff through reduced working hours and increased pay alone.^126^ Excessive temporal commitment on behalf of hospital doctors is evidently a problem—not least for those with caring responsibilities—but it might not be the sole barrier to an emotionally healthy workforce.^127^ Evidence from earlier in the twentieth century suggests that solutions to depleted staff well-being might be more intangible and therefore tricky to implement. How, for example, do you cultivate collegiality among hospital or operating theater staff? And what might have worked for a 1960s workforce is less likely to prove effective today. The purpose of this article is not to provide new policies, or draw straightforward parallels between then and now, but to suggest alternative avenues for investigation. My intention is to prompt a more nuanced and historically engaged discussion about the role of feelings in the clinical[Other P-100] workplace and call on historians, practitioners, and policy makers to consider the emotional landscape of the hospital, both past and present.[Other P-101]

## Footnotes

1
ColcockBentley P.Report to the James IV Association of Surgeons1964

2 James IV Association of Surgeons Records.

3 Ibid.

4
BrownMichaelSurgery, Identity and Embodied Emotion: John Bell, James Gregory and the Edinburgh 'Medical War,'History104201919413082810510.1111/1468-229X.12720PMC6378620

5 Hugh Dudley, Excerpts from Tributes to Sir John Bruce (1905–1975) by the Hon. David A. Balfour, Adam N. Smith and Professor Hugh Dudley, Word from the James IV Association of Surgeons, Inc., Spring 1976, box 1, folder 2, Minutes, etc. 1971, 1975–1982, James IV Association of Surgeons Records.

6
ReddyWilliam M.The Navigation of Feeling: A Framework for the History of EmotionsCambridgeCambridge University Press2009

7
BrownMichaelSurgery and Emotion: The Era Before AnaesthesiaThe Palgrave Handbook of the History of SurgerySchlichThomasLondonPalgrave Macmillan201732724829419977

8
PrenticeRachelBodies in Formation: An Ethnography of Anatomy and Surgery EducationDurham, N.C.Duke University Press2012

9
LanghamerClaireFeelings, Women and Work in the Long 1950sWomen's Hist. Rev.262017779279

10
SchlichPalgrave Handbook of the History of Surgery7

11
Schlich'One and the Same the World Over': The International Culture of Surgical Exchange in an Age of Globalization, 1870–1914J Hist. Med.& Allied Sci.7120162472702688894210.1093/jhmas/jrw003PMC4986222

12
GavrusDeliaMen of Dreams and Men of Actions: Neurologists, Neurosurgeons and the Performance of Professional Identity, 1925–1950Bull. Hist. Med.85201157922155191710.1353/bhm.2011.0020 KernahanPeterSurgery Becomes a Specialty: Professional Boundaries and SurgerySchlichPalgrave Handbook of the History of Surgery795113 SchlichThomasSurgery, Science and Industry: A Revolution in Fracture Care, 1950–1990sBasingstokePalgrave2002 Degrees of Control: The Spread of Operative Fracture Care with Metal Implants. A Comparative Perspective of Switzerland, East Germany, and the USA, 1950s–1990sInnovations in Health and Medicine: Diffusion and Resistance in the Twentieth CenturyStantonJennyLondonRoutledge2002106125

13
SchlichThomasIntroduction: What Is Special about the History of SurgerySchlichPalgrave Handbook of the History of Surgery712412

14
SchlichDegrees of Control12107

15 Dudley, Excerpts from Tributes to Sir John Bruce (n. 5).

16 Ibid.

17
ColcockReport to the James IV Association of Surgeons1

18
StephensonHugh E.Undergraduate Surgical Teaching in the United Kingdom and IrelandSurgery65196432133314213827

19
FitzharrisLindseyThe Butchering Art: Joseph Lister's Quest to Transform the Grisly World of Victorian MedicineLondonPenguin2017

20 Adam N. Smith, Excerpts from Tributes to Sir John Bruce (1905–1975) by the Hon. David A. Balfour, Adam N. Smith and Professor Hugh Dudley, Word from the James IV Association of Surgeons, Inc., Spring 1976, box 1, folder 2, Minutes, etc. 1971, 1975–1982, James IV Association of Surgeons Records.

21
BrownMichaelRobert Liston, the 'Fastest Knife in the West End'? Part One: Historical Myth-MakingSurgery & Emotion062019http://www.surgeryandemotion.com/blog/view/robert-liston-the-fastest-knife-in-the-west-end-part-1-historical-myth-making

22 Dudley, Excerpts from Tributes to Sir John Bruce (n. 5).

23
StephensonUndergraduate Surgical Teaching in the United Kingdom and Ireland1832214213827

24 Eric W. Fonkalsrud, "Report to the James IV Association of Surgeons," Surgical Traveller for 1971 to Great Britain and Norway, James IV Association of Surgeons Records.

25
ColcockReport to the James IV Association of Surgeons1

26
SablesDavidIndustry, Heritage, the Media, and the Formation of a British National Cultural MemoryInt. J. Hist. Archaeol.2120179781010994

27
RedmanN. R.TobyJ. P.PeniketJ. B.Hospital Junior Doctors: Survey at Northampton General HospitalBrit. Med. J.31969522525525580134910.1136/bmj.3.5669.522PMC1984269

28
T.C. Graves, B.Sc., M.D., F.R.C.S., M.R.C.V.S.Brit. Med. J.119641711

29
KernahanSurgery Becomes a Specialty1295

30
Schlich'One and the Same the World Over'11

31
ColcockReport to the James IV Association of Surgeons1

32 Minutes of the Annual Meeting of Members, James IV Association of Surgeons, Inc. November 8, 1976, Surgical Travellers for 1977, James IV Association of Surgeons Records.

33 R. A. Macbeth, "Report on the James IV Association of Surgeons Inc. Surgical Travelership to the United Kingdom, Eire, Denmark and Sweden," James IV Association of Surgeons Records.

34
ColcockReport to the James IV Association of Surgeons1

35 Ibid.

36 Folkert O. Belzer, "Report to the James IV Association of Surgeons," Surgical Traveller for 1977 to England, Poland, the Netherlands, and Sweden, James IV Association of Surgeons Records.

37 Ibid.

38 Ibid.

39 E. G. Muir, "Report on a Visit to the United States and Canada as James IV Travelling Professor, 1961," James IV Association of Surgeons Records.

40 Edward G. Tuckwell, "Report of the First Ian Aird Surgical Traveller, United States and Canada 1963," James IV Association of Surgeons Records.

41 Terence Kennedy, "Visit to the USA as James IV Surgical Traveller from Ireland," James IV Association of Surgeons Records.

42
ColcockReport to the James IV Association of Surgeons1

43 Ibid.

44
MacbethRonaldMarried Quarters in HospitalBrit. Med. J.119631673167416741395198410.1136/bmj.1.5341.1344-aPMC2123911

45
MacbethRonaldMarried Quarters in HospitalBrit. Med. J.11963167410.1136/bmj.1.5341.1344-aPMC212391113951984

46 Minutes of the Meeting of the Board of Directors, James IV Association of Surgeons, Inc., September 9, 1983, "Discussion and Selection of Candidates for Election to Active Membership," James IV Association of Surgeons Records.

47 Ibid.

48
ColcockReport to the James IV Association of Surgeons1

49 Ibid.

50 Minutes of an Interim Meeting of the North American Members, James IV Association of Surgeons, Inc., October 23, 1979, James IV Association of Surgeons Records.

51 Ibid.

52 Ibid.

53 Ibid.

54 Victor Riddell, "Visit to India as James IV Surgical Traveller 1963," James IV Association of Surgeons Records.

55 Ibid.

56 Ibid.

57
BivinsRobertaContagious Communities: Medicine, Migration, and the NHS in Post War BritainOxfordOxford University Press2015

58 Letter from Mr. P. Harding to A. Merrison, March 21, 1976, National Archive, BS6/10.

59 Ibid.

60
StephensonUndergraduate Surgical Teaching in the United Kingdom and Ireland1832214213827 SimpsonJulian M.SnowStephanie J.EsmailAneezProviding 'Special' Types of Labour and Exerting Agency: How Migrant Doctors Have Shaped the UK's National Health ServiceDoctors Beyond Borders: The Transnational Migration of Physicians in the Twentieth CenturyMonnaisLaurenceWrightDavidTorontoUniversity of Toronto Press2016

61
AlamEramCold War Crises: Foreign Medical Graduates Respond to US Doctor Shortages, 1965–1975Soc. Hist. Med.332018132151136

62
AlamEramThe Care of Foreigners: A History of South Asian Physicians in the United States, 1965–2016Ph.D. diss.University of Pennsylvania20163https://repository.upenn.edu/edissertations/2161

63
MonnaisWrightDoctors Beyond Borders60230256231

64
StephensonUndergraduate Surgical Teaching in the United Kingdom and Ireland1832214213827

65
ColcockReport to the James IV Association of Surgeons1

66 Ibid.

67
FergusonColin C.Summary of a Visit to England, Scotland and Ireland as a James IV Surgical Traveller1965James IV Association of Surgeons Records14234227

68
GriffithsJohn D.Report on a Visit to United States and Canada1971James IV Association of Surgeons Records

69
StephensonUndergraduate Surgical Teaching in the United Kingdom and Ireland1832414213827

70 Minutes of the Annual Meeting of Members, James IV Association of Surgeons, Inc., October 25, 1988, James IV Association of Surgeons Records.

71
BrockClaireBritish Women Surgeons and Their Patients, 1860–1918CambridgeCambridge University Press2017

72
StephensonUndergraduate Surgical Teaching in the United Kingdom and Ireland1832614213827

73
StephensonUndergraduate Surgical Teaching in the United Kingdom and Ireland1832614213827

74
StephensonUndergraduate Surgical Teaching in the United Kingdom and Ireland1832414213827

75
JohnsonP. A.Married QuartersBrit. Med. J.11967507

76
StephensonUndergraduate Surgical Teaching in the United Kingdom and Ireland1832614213827

77
FergusonSummary of a Visit to England, Scotland and Ireland67

78 Ibid.

79
PressleyHildaStaff Nurses in LoveLondonMills & Boon19629

80
OxtobyKathyLove on the Wards: Relationships between DoctorsBrit. Med. J.3512015

81
KennedyVisit to the USA as James IV Surgical Traveller from Ireland41

82 https://researchbriefings.files.parliament.uk/documents/SN04252/SN04252.pdf.

83
MintonJohn PeterReport to the James IV Association of Surgeons1975James IV Association of Surgeons Records

84 HC Deb, March 24, 1976, vol. 908, cc190-1W, https://api.parliament.uk/historic-hansard/written-answers/1976/mar/24/doctors-pay.

85 UK Retail Price Index inflation figures are based on data from Gregory Clarke, https://measuringworth.com/datasets/ukearncpi/.

86 HC Deb, April 18, 1975, vol. 890, c176W, https://api.parliament.uk/historic-hansard/written-answers/1975/apr/18/manual-workers-average-wage.

87
MintonReport to the James IV Association of Surgeons83

88
FonkalsrudReport to the James IV Association of Surgeons245057656

89
StephensonUndergraduate Surgical Teaching in the United Kingdom and Ireland1832414213827

90
HugginsMikeSport and the British Upper Classes *c*.1500–2000: A Historiographic OverviewSport Hist.282008364388

91
StephensonUndergraduate Surgical Teaching in the United Kingdom and Ireland1832414213827

92
StephensonUndergraduate Surgical Teaching in the United Kingdom and Ireland1832414213827

93
StephensonUndergraduate Surgical Teaching in the United Kingdom and Ireland1832414213827

94
AtkinsHedleyThe Surgeon's CraftManchesterManchester University Press196530

95
StephensonUndergraduate Surgical Teaching in the United Kingdom and Ireland1832614213827

96
StephensonUndergraduate Surgical Teaching in the United Kingdom and Ireland1832614213827

97
ClarkeAlan M.Report to James IV Association of Surgeons1970James IV Association of Surgeons Records

98
StephensonUndergraduate Surgical Teaching in the United Kingdom and Ireland1832314213827

99
FonkalsrudReport to the James IV Association of Surgeons245057656

100
SmithAdam N.Report on a Medical Tour of the United States of America1963James IV Association of Surgeons Records

101 Ibid.

102
StephensonUndergraduate Surgical Teaching in the United Kingdom and Ireland1833314213827

103
FonkalsrudReport to the James IV Association of Surgeons245057656

104 Ibid.

105
StephensonUndergraduate Surgical Teaching in the United Kingdom and Ireland1833014213827

106
StephensonUndergraduate Surgical Teaching in the United Kingdom and Ireland1832614213827

107
StephensonUndergraduate Surgical Teaching in the United Kingdom and Ireland1832332414213827

108
TuckwellReport of the First Ian Aird Surgical Traveller40

109
DayGeorgePersonal ViewBrit. Med. J.31968182

110
DayGeorgePersonal ViewBrit. Med. J.31968182

111
DayGeorgePersonal ViewBrit. Med. J.31968182

112 Interview with male surgeon born in 1944; interviewed by the author, January 18, 2018.

113 Ibid.

114
SmithReport on a Medical Tour of the United States of America100

115 Ibid.

116 Ibid.

117 Ibid.

118 Ibid.

119 Ibid.

120
NoelOlivier F.Ethnic and Gender Diversity Comparison between Surgical Patients and Caring SurgeonsPlast. Reconstr. Surg. Glob. Open8202010.1097/GOX.0000000000003198PMC764751733173701 JosephJ. P.BAME Underrepresentation in Surgery Leadership in the UK and Ireland in 2020: An Uncomfortable TruthRoy. Coll. Surg. Bull.1662020232233

121
Freedman-WeissMollie R.Understanding the Barriers to Reporting Sexual Harassment in Surgical TrainingAnn. Surg.271201960861310.1097/SLA.000000000000329530946072

122
RyanRenaeBlackKirstenCookVictoriaThe Hidden Curriculum in Surgery Says It's Not for WomenThe Conversation0822016https://theconversation.com/the-hidden-curriculum-in-surgery-says-its-not-for-women-63109

123
BalchCharles M.Stress and Burnout among Surgeons: Understanding and Managing the Syndrome and Avoiding the Adverse ConsequencesArch. Surg.14420093713761938065210.1001/archsurg.2008.575 GeradaClareClinical Depression: Surgeons and Mental IllnessBull. Roy. Coll. Surg. England2017260262

124
British Medical AssociationSupporting Health and Wellbeing at Work2018https://www.bma.org.uk/media/2076/bma-supporting-health-and-wellbeing-at-workoct-2018.pdf

125
Arnold-ForsterAgnesResilience in SurgeryBrit. J. Surg.10720203323333212948410.1002/bjs.11493PMC7078967

126
ForsythRachael O.SuttieStart A.Enhancing Junior Doctors' Working LivesSurgery3820206076113290469310.1016/j.mpsur.2020.07.002PMC7456575

127
LachishShellyFactors Associated with Less-Than-Full-Time Working in Medical Practice: Results of Surveys of Five Cohorts of UK Doctors, 10 Years after GraduationHum. Resources Health14201610.1186/s12960-016-0162-3PMC506489927737659

